# Bridger: a new framework for *de novo* transcriptome assembly using RNA-seq data

**DOI:** 10.1186/s13059-015-0596-2

**Published:** 2015-02-11

**Authors:** Zheng Chang, Guojun Li, Juntao Liu, Yu Zhang, Cody Ashby, Deli Liu, Carole L Cramer, Xiuzhen Huang

**Affiliations:** School of Mathematics, Shandong University, Jinan, Shandong 250100 China; College of Computer Science and Technology, Jilin University, Changchun, Jilin 132000 China; Molecular Biosciences Program, Arkansas State University, Jonesboro, Arkansas 72401 USA; Department of Biochemistry and Molecular Biology, University of Georgia, Athens, Georgia 30602 USA; Institute of Bioinformatics, University of Georgia, Athens, ᅟ, Georgia 30602 USA; Arkansas Bioscience Institute and Department of Biological Sciences, Arkansas State University, Jonesboro, Arkansas 72401 USA; Department of Computer Science, Arkansas State University, Jonesboro, Arkansas 72401 USA

## Abstract

We present a new *de novo* transcriptome assembler, Bridger, which takes advantage of techniques employed in Cufflinks to overcome limitations of the existing *de novo* assemblers. When tested on dog, human, and mouse RNA-seq data, Bridger assembled more full-length reference transcripts while reporting considerably fewer candidate transcripts, hence greatly reducing false positive transcripts in comparison with the state-of-the-art assemblers. It runs substantially faster and requires much less memory space than most assemblers. More interestingly, Bridger reaches a comparable level of sensitivity and accuracy with Cufflinks. Bridger is available at https://sourceforge.net/projects/rnaseqassembly/files/?source=navbar.

**Electronic supplementary material:**

The online version of this article (doi:10.1186/s13059-015-0596-2) contains supplementary material, which is available to authorized users.

## Background

RNA-seq is a powerful technique for collecting gene-expression data at a whole transcriptome level with unprecedented sensitivity and accuracy [1-4]. Compared with microarray chips and EST sequencing, RNA-seq achieves the single-nucleotide resolution, has a substantially higher dynamic range, and allows reliable identification of rare transcripts and alternative splicing [2-5]. However, the sequence reads obtained from RNA sequencing tend to be very short [6], hence posting tremendous computational challenges to reconstruct the full-length transcripts from the reads.

At first glance, an RNA-seq assembly problem is similar to the problem of genome assembly. However short-read genome assemblers, such as Velvet [7], ABySS [8], and ALLPATHS [9], cannot be directly applied to transcriptome assembly, due to the following reasons: (1) DNA sequencing depth is expected to be the same across a genome while the depths of the sequenced transcripts may vary by several orders of magnitude [10]; and (2) due to alternative splicing, a transcriptome-assembly problem is more complex than a linear problem as in the case of genome assembly, generally requiring a graph to represent the multiple alternative transcripts per locus [11]. These characteristics have made the transcriptome assembly problem computationally more challenging than the genome assembly problem.

A number of RNA-seq based transcriptome assemblers have been developed in the past few years. They fall into two general categories: reference-based and *de novo* assembly approaches [10,11]. The basic idea of a reference-based approach, such as Cufflinks [12] and Scripture [13], has the following steps. First, RNA-seq reads are aligned to a reference genome using a splice-aware aligner such as Blat [14], TopHat [15], SpliceMap [16], MapSplice [17], or GSNAP [18]. Second, overlapping reads from each locus are merged to build a graph representing all possible splicing isoforms. Finally, full-length splicing isoforms are recovered by traversing the graph. This strategy is used only when a high-quality reference genome is available.

*De novo* assembly is used when no reliable reference genome is available, including situations when dealing with human cancer transcriptomes as their genomes tend to be considerably altered compared to the corresponding healthy genomes of the same patients. A number of *de novo* assemblers, such as ABySS [19], SOAPdenovo [20], Oases [21], and SOAPdenovo-Trans [22] have been developed, some of which do not work well since they rely on the key ideas of genome-assembly methods. Trinity [11] is the first method designed specifically for transcriptome assembly. It assembles a transcriptome by first extending individual RNA-seq reads into longer contigs, building many *de Bruijn* graphs from these contigs, and then deriving all the splicing-isoform-representing paths in each graph. While Trinity has greatly improved the assembly performance over the previous *de novo* assemblers, it has a number of limitations that need improvements. For example, Trinity used an exhaustive enumeration algorithm to search for isoform-representing paths in a *de Bruijn* graph, which makes the algorithm highly sensitive to splicing isoforms but suffers from having high false positives. We believe that by identifying an optimal set of potential isoform-representing paths, one can reduce the false positive predictions significantly. In addition, all existing *de novo* assemblers, Trinity included, use only paired-reads to resolve assembly ambiguities, particularly those relevant to alternative splicing, instead of using more direct evidences to support their predicted transcripts, which tend to give rise to false predictions. Actually the information that different locations of the same transcript should have the same or similar levels of sequence depth provides a direct and strong constraint on the assembly problem. While it has been noted that such information will be useful for the accurate assembly of a transcriptome [11], none of the current *de novo* assemblers have included this information in a rigorous manner, due to the technical challenge involved. Hence how to integrate such information into a *de novo* assembly program remains an open problem.

As of now, all the existing *de novo* assemblers use a *de Bruijn* graph to represent the assembly problem, which processes each sequence into a set of overlapping substrings of length *k* bps, called *k-mers*, where *k* is a parameter, and recover the splicing isoforms from the graph [23]. Generally speaking, larger *k* values tend to perform better on transcripts with high gene-expression levels or longer contigs, while smaller *k* values perform better on transcripts with low gene-expression levels or shorter contigs. It seems unlikely that a single *k* value will yield an optimal overall assembly. Hence some assemblers, such as Trans-ABySS [23], Oases-M [21] (multiple-*k* version of Oases), and IDBA-Tran [24], use a multiple-*k* strategy, in which multiple assemblies using different *k* values are merged to get a higher sensitivity, but at the cost of introducing more false positive transcripts.

In this paper, we present a new assembler, Bridger, aiming to build a bridge between the key ideas of two popular assemblers, the reference-based assembler Cufflinks [12] and *de novo* assembler Trinity [11]. Specifically, we have generalized the main techniques employed by Cufflinks to overcome the limitations of Trinity, hence to develop a more general *de novo* assembler better than the state of the art. We have tested Bridger on two standard RNA-seq datasets, one dog and one human, and on one strand-specific mouse RNA-seq data. In each case, Bridger assembled more reference transcripts than the other *de novo* assemblers, while reporting 10,000 to 30,000 fewer candidate transcripts, which greatly reduced the false-positive assemblies. In addition, Bridger runs much faster and requires less memory space than most of compared methods, and exhibits competitive CPU time. The performance of Bridger is even comparable with the reference-based assembler Cufflinks in both sensitivity and accuracy. In addition, a multiple-*k* version of Bridger, Bridger*-M*, can further improve the assembly sensitivity by merging assemblies from different *k* values.

## Methods

First recall the definition of a splicing graph introduced by Heber *et al.* in 2002 [25]. A splicing graph of a gene is a directed acyclic graph, whose nodes correspond to exons and edges represent splicing junctions, where splicing events take place. Bridger will reconstruct different alternative splicing transcripts by considering only the splicing junctions, which has been demonstrated to be feasible by a recent research paper [26].

**Figure 1:**
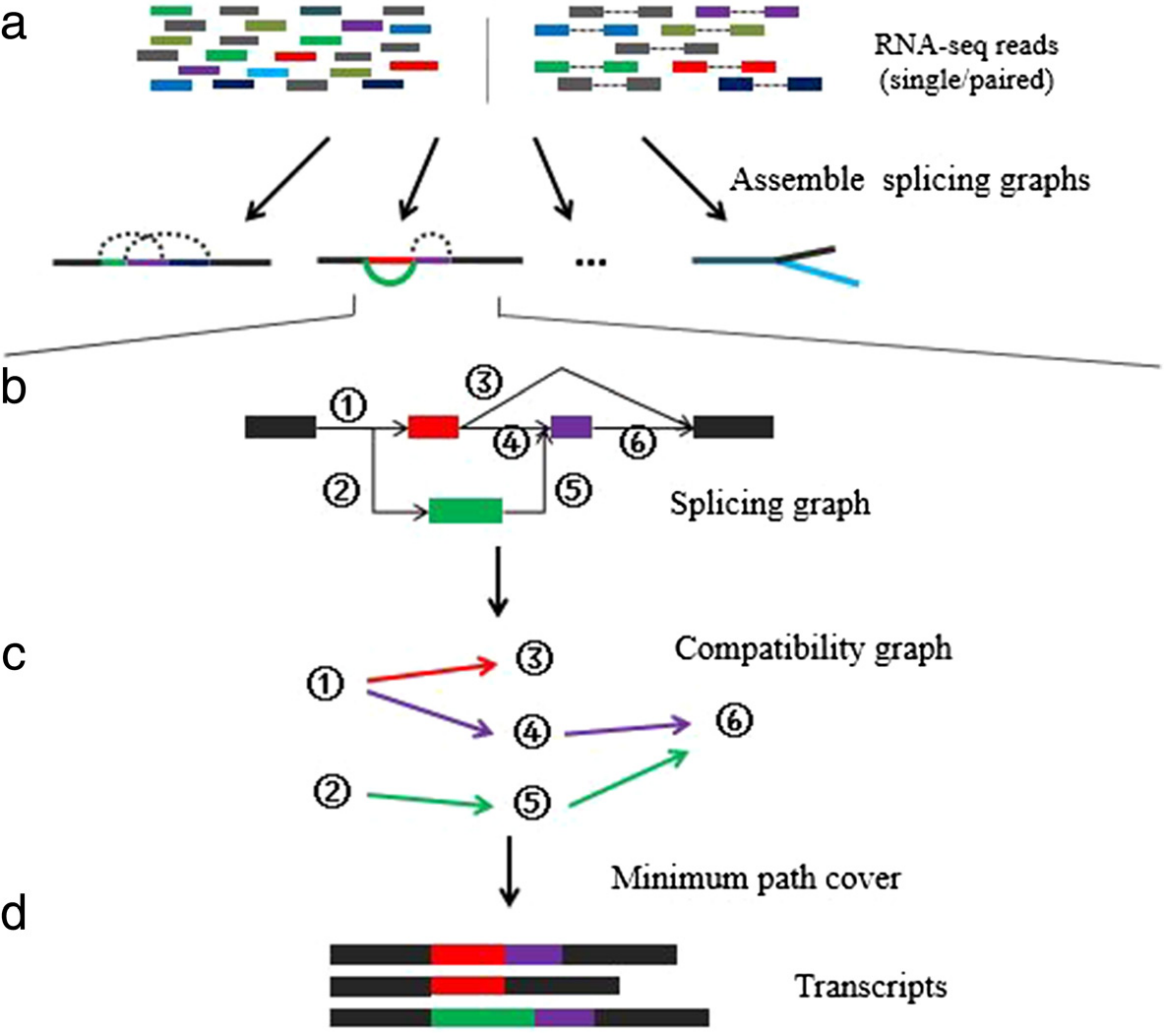
**Flowchart of Bridger. (a)** The algorithm takes RNA-seq reads (single or paired) to assemble splicing graphs, each of which provides a complete representation of all alternative splicing transcripts for each locus. (b-d) Each splicing graph is processed independently. **(b)** Each edge in a splicing graph represents one splice junction. In this example, edges 1 and 3 are compatible, while edges 3 and 4 are not compatible. **(c)** A compatibility graph. **(d)** A minimum path cover model is applied to recover a minimal set of transcripts that could be tiled together through overlapping sequence reads and ‘explain’ all observed junctions in a splicing graph.

Bridger builds splicing graphs for all genes encoded in the genome based on the given RNA-seq data. In an ideal situation, the constructed graphs would have a one-to-one correspondence to all the (expressed) genes. Sometimes it is not the case due to homologous genes and low sequence depths for some genes, but it will not seriously affect us to recover full-length transcripts of individual genes even if some splicing graphs cover multiple genes or only parts of a gene. Hence, we assume without loss of generality that each created graph represents RNA-seq data of one gene. Bridger uses a rigorous mathematical model, called the minimum path cover, to search for a minimal set of paths (transcripts) that are supported by the provided RNA-seq reads and can explain all the observed splicing events of the created graph. A flowchart of the Bridger algorithm is given in Figure 1.

### Efficient construction of splicing graphs

As in Trinity, Bridger first constructs a hash table from all RNA-seq reads. For each *k*-mer occurring in the reads, the hash table records the abundance of that *k*-mer and the IDs of all the reads containing the *k*-mer. The same removal process of erroneous *k*-mers (induced from sequencing errors) in Trinity is employed in Bridger [11]. Bridger constructs splicing graphs as follows:

1. Select a most frequent *k*-mer as the main contig of the initial splicing graph, excluding both low-complexity (Shannon’s entropy *H* <1.5) and singleton (appearing only once) *k*-mers;
2. Extend the main contig at the 5′ end through finding a most frequent unused *k*-mer, whose (*k-1*) suffix is identical to the (*k-1*) prefix of the main contig, and extending it by appending the first nucleotide of the *k*-mer as the new 5′ end; and do the same to the 3′ end of the main contig when possible; continue this step until the main contig could not be further extended in either end;
3. Further extend the main contig using a similar idea in SOAPdenovo-Trans [22] when paired-read information is available (see Additional file 1: Methods and Figure S1), which is inspired by how to connect several short contigs into a longer one by finding supporting paired-reads during the scaffolding and finishing step of genome-assembly [27,28];
4. For each *k*-mer in the current splicing graph, check if it has at least one alternative extension not existing in the current graph (for example, the left red *k*-mer ATCAG in Figure 2a). Such a k-mer is called a bifurcation *k*-mer. For each bifurcation k-mer, we keep extending it until either encountering an already used *k*-mer (the red 5-mer CTAGC in Figure 2a) or no further extension by using steps (1) to (3). If the former occurs, then a new bifurcation *k*-mer is found (for example, the right red *k*-mer CTAGC in Figure 2a), and update the current splicing graph by merging their matched (*k*-1) nucleotides (see Figure 2b). If the latter occurs, a potential branch point is identified, where paired-read information, if available, as well as some criteria, are used to check if it is a true branch point that should be added into the current graph or it is a false one resulted from paralogous genes or sequencing errors (see Additional file 1: Methods and Figure S2); Repeats this until no bifurcation *k*-mer exists;

**Figure 2:**
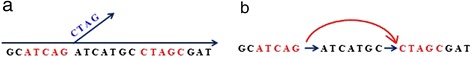
**Splicing graph construction. (a)** Splicing graph after branch extension. The red *k*-mer (k = 5) ATCAG on the left is a bifurcation 5-mer because there is an unused 5-mer TCAGC in the hash table that provides an alternative extension. Extend this 5-mer to a new contig until it cannot be further extended. We check the last 4-mer of this branch to see if there is a matching 4-mer in the current splicing graph. If so, another bifurcation 5-mer is found (for example, the red 5-mer CTAGC). **(b)** A modified splicing graph by merging the k-1 overlapping nucleotides (4-mer CTAG) and adding a new directed edge between two bifurcation *k*-mers.
5. Now a splicing graph is constructed; all *k*-mers used in this splicing graph will be marked to indicate their lower priority of being reused for extension in the future;
6. Remove edges due to sequencing errors through aligning reads back to splicing graphs using a similar idea in Trinity [11] and IDBA-Tran [24] (see Additional file 1: Methods for the detailed criteria), which can be done efficiently based on the hash table;
7. Select a most frequent unused *k*-mer as a new seed, repeats steps (1) to (6) until the entire hash table has been exhausted.

Note that the splicing graph constructed here is different from the contracted *de Bruijn* graph even though there is a kind of correspondence between them. First, a splicing junction corresponds to an edge in the splicing graph, while in the contracted *de Bruijn* graph, a splicing junction corresponds to a node, the same as an exon does (Additional file 1: Figure S7). Second, the splicing graph can be guaranteed to be acyclic, which is necessary to use the minimum path cover model, while the contracted *de Bruijn* graph might have cycles. Finally, the *de Bruijn* graph usually suffers from a problem that the first graph built from the hash table is very huge because many genes are mixed together by their sharing *k*-mers. However, the splicing graph constructed here could keep a size small by using paired-read information to check if a new branch should be added (Additional file 1: Method), which makes transcript discovery much easier.

Splicing graphs provide a natural and lossless representation of all the (alternatively) splicing isoforms in a transcriptome. By analyzing the structure of splicing graphs, we discovered that a transcript reconstruction can be achieved based solely on splicing junctions in splicing graphs; hence recovering each full-length transcript from a splicing graph can be viewed as finding the most likely combination of the junctions of this graph.

### Construction of compatibility graphs

To recover all transcripts encoded in a splicing graph, Bridger constructs a directed acyclic graph *C*, called a compatibility graph, in which each edge (junction) of the splicing graph is represented as a node and a directed edge (*x*, *y*) is placed between nodes *x* and *y* if *x* and *y* are compatible, that is, they correspond to consecutive edges (one goes into the exon from which the other gets out) in the splicing graph, implying that they may come from the same spliced isoform. It is not difficult to see that each transcript to be reconstructed has to correspond to a directed path of the compatibility graph while the reverse is not necessarily true. To make sure a directed path that we will construct has to correspond to a transcript encoded in the genome, we need to find a way to compel all nodes in the path come from the same transcript. To do so, we need to weight each compatibility graph as follows: for each node *x* in a compatibility graph, its corresponding junction edge in the splicing graph is an arc (*e’*, *e”*), with arc tail *e’* and arc head *e”*, we assign out-weight *W*_*x,o*_ and in-weight *W*_*x,i*_ to the node *x*, where *W*_*x,o*_ is defined as the ratio between the number of reads (or paired reads for paired-end sequencing) spanning the junction (*e’*, *e”*) and the total number of reads that span all the junctions that have the same arc tail (or 5′ end exon) *e’* with (*e’*, *e”*) in the splicing graph; and *W*_*x,i*_ is the ratio between the number of reads spanning the junction (*e’*, *e”*) and the total number of reads that span all the junctions that have the same arc head (or 3′ end exon) *e”* with (*e’*, *e”*) in the splicing graph.

### Recovery of full-length transcripts

It should be noted that the compatibility graphs can play the same role as the overlap graphs in Cufflinks, which are built from reads aligned to the reference genome. The rationale is as follows. In Cufflinks [12], an overlap graph is defined over the provided RNA sequence fragments, each represented as a node, in which two fragments are connected by an edge if they are compatible. All the full-length transcripts were reconstructed from a set of mutually incompatible fragments. Here, we replace the compatibility between fragments in Cufflinks by the compatibility between splicing junctions, and find a set of mutually incompatible junctions, each of which could be extended to be a transcript. However, the compatibility graph defined here is tremendously smaller in size than overlap graph used in Cufflinks.

Bridger recovers all the full-length transcripts by employing the same techniques as Cufflinks but on the compatibility graphs. Specifically, we first compute the transitive closure *G* of the compatibility graph *C*. For the presentation clarity, we define a bipartite graph, called a reachability graph, over *G*. For each node *x* in *G*, define two nodes *L*_*x*_ and *R*_*x*_ with *L*_*x*_ in the left partition and *R*_*x*_ in the right partition of the bipartite graph; any two nodes *L*_*x*_ and *R*_*y*_, one from each partition, have an edge between them if and only if there is a directed edge (*x*, *y*) in *G*; and each edge (*L*_*x*_*, R*_*y*_) has a weight *W*_*x,y*_ *= − log(1 - |W*_*x,i*_*- W*_*y,o*_*|)*, where *W*_*x,i*_ and *W*_*y,o*_, respectively, are in-weight of node *x* and out-weight of node *y* in *C*, which is intended to reflect the prediction confidence that two corresponding junctions are from different transcripts. We then compute a min-cost maximum cardinality matching *M* on this bipartite graph. Based on Dilworth’s Theorem (see Additional file 1: Theorem 1) [29], a minimum path cover of the compatibility graph with the minimum cost can be constructed from this matching (see Additional file 1: Methods). Note that each node in the compatibility graph corresponds to two exons connected by one junction edge in the splicing graph, so the minimum path cover of the compatibility graph can be converted into a path cover of the splicing graph, which meets the following criteria: (1) each junction of the splicing graph is consistent with at least one transcript; (2) every transcript is tiled by sequence reads; (3) the cardinality of the obtained set of transcripts is minimized subject to (1) and (2).

For all the predicted transcripts, one additional filtering step is used to remove those predictions that are not supported by tiled paired read data with coverage no less than a cutoff *c* (*c* = 2) (see Additional file 1: Figure S3e). It is worth noting that the sequence depth information has been implicitly considered in the node weights of the compatibility graph. Specifically, the smaller the value *|W*_*x,i*_*- W*_*y,o*_*|* is, the higher the probability that the two junctions *x* and *y* fall into the same transcript is. For junctions from the same transcript, they should have similar expression levels and hence a similar sequence depth.

## Results and discussion

Bridger has been compared to ABySS (version 1.3.4) [19], Trans-ABySS (version 1.4.4) [23], Trinity (version 2012-10-05) [11], Velvet (version 1.2.01) + Oases (version 0.2.02) [7,21], SOAPdenovo-Trans (version 1.01) [22] and IDBA-Tran (version 1.1.1) [24] on the following datasets and using parameters outlined below. Multiple *k* versions of Oases and Bridger are named as Oases-M and Bridger-M in order to differentiate them from their single k versions. The reference-based assembler Cufflinks (version 2.0.2) [12] is also included as a benchmark for *de novo* assembly.

- *Datasets*: three RNA-seq datasets were used: two standard (non-strand specific) Illumina datasets from dog and human, and one strand-specific dataset from mouse. The mouse and human data are selected because they were the test data in Oases [21] and Trinity [11]. It is more convincing to compare with these methods on the datasets used in their papers. The dog data were produced in a recent study by Liu, *et al*. [30], with about 31 million 50 bp paired-end reads with an insert size of 130 bp sequenced. The human data (Accession Codes: SRX011545 and SRX011546) were collected on human CD4 T cells [31], with 50 million paired-end reads of length 45 bp with an insert size of 200 bp, which we downloaded from the DDBJ SRA database. The mouse data (Accession Code: SRX062280 in the DDBJ SRA database) were collected by Grabherr, *et al*. [11], with 53 million 76 bp paired-end reads with an insert size of 300 bp sequenced from C567BL/6 mouse primary immune dendritic cells.
- *Parameter setup*: the following parameters are used for each program: ABySS: ‘abyss-pe c = 2 E = 0 j = 6 in = “left.fq right.fq”’, k = 25 for dog and human data while k = 33 for mouse data; Trinity: ‘--CPU 6 --bflyHeapSpaceMax 10G --bflyGCThreads 4’ for human and dog data; and ‘--SS_lib_type RF’ for the strand-specific mouse data; Oases : ‘-ins_length 200 -cov_cutoff 2 -edgeFractionCutoff 0.05’, k = 25 for dog and human data while k = 31 and ‘-strand_specific’ for mouse data; Bridger: k = 25 for dog and human data while k = 31 and ‘-- SS_lib_type RF’ for mouse data; Cufflink was run using default parameters; Trans-ABySS, Oases-M, and IDBA-Tran were run by setting *k*-mer length *k* to 21, 25, 29, 33, 37 on the dog and human data, and 25, 29, 33, 37, 43 on mouse data (see Additional file 1: Note); and Bridger-M was run by setting *k* to 21, 23, 25, 27, 29 on the dog and human data, and 23, 25, 27, 29, 31 on mouse data. For multiple *k* assemblers, all assembly results using different *k* values were merged using Oases. For fair comparison of run time and required memory space, we used k = 25 for all the programs. Note that *k* = 25 is only used for the run time and memory usage comparison, while an optimal *k* is found for each assembler for sensitivity and accuracy comparison in the case that *k* = 25 is not optimal (Additional file 1: Note, Figure S5, Figure S6, and Table S6). Non-default parameters are used for Oases because these parameters perform better than default (Additional file 1: Table S7). All the assemblies were performed on a server with 512 GB of RAM. Only transcripts with no less than 200 bases were used for downstream analysis.
- *Comparing to reference transcripts*: the known transcripts of dog were downloaded from UCSC. The human and mouse known transcripts were downloaded from Ensembl Genome Browser. All the assembled transcripts were compared with these reference transcripts using BLAT [14], using 95% identity as the cutoff. If one assembled transcript can full-length cover one reference transcript with at least 95% sequence identity and at most 1% indels, we say this reference transcript is full-length reconstructed. Similarly, ≥80% length reconstructed reference transcripts were defined as those reference transcripts having at least 80% of its sequence was covered by some assembled transcript and at most 1% indels. The indel cutoff is used mainly to avoid the potential problem of over-estimating consistencies between predicted transcripts and the reference transcripts.

### Comparing Bridger to other methods

We have considered the following measures in the performance comparison with other state-of-the-art assemblers: the number of reference transcripts reconstructed to full-length by each method, referred to as sensitivity, and the rate between the number of full-length reconstructed reference transcripts and the number of candidate transcripts, referred to as accuracy. The sensitivity measure used here may have slightly different meaning from its typical meaning, which is the ratio between the number of full-length reconstructed transcripts and the number of all expressed transcripts in the given data, because the denominator of this ratio is unknown and is not easy to estimate accurately. The accuracy measure indicates the power of detecting the most reference transcripts using the least candidates.

**Figure 3:**
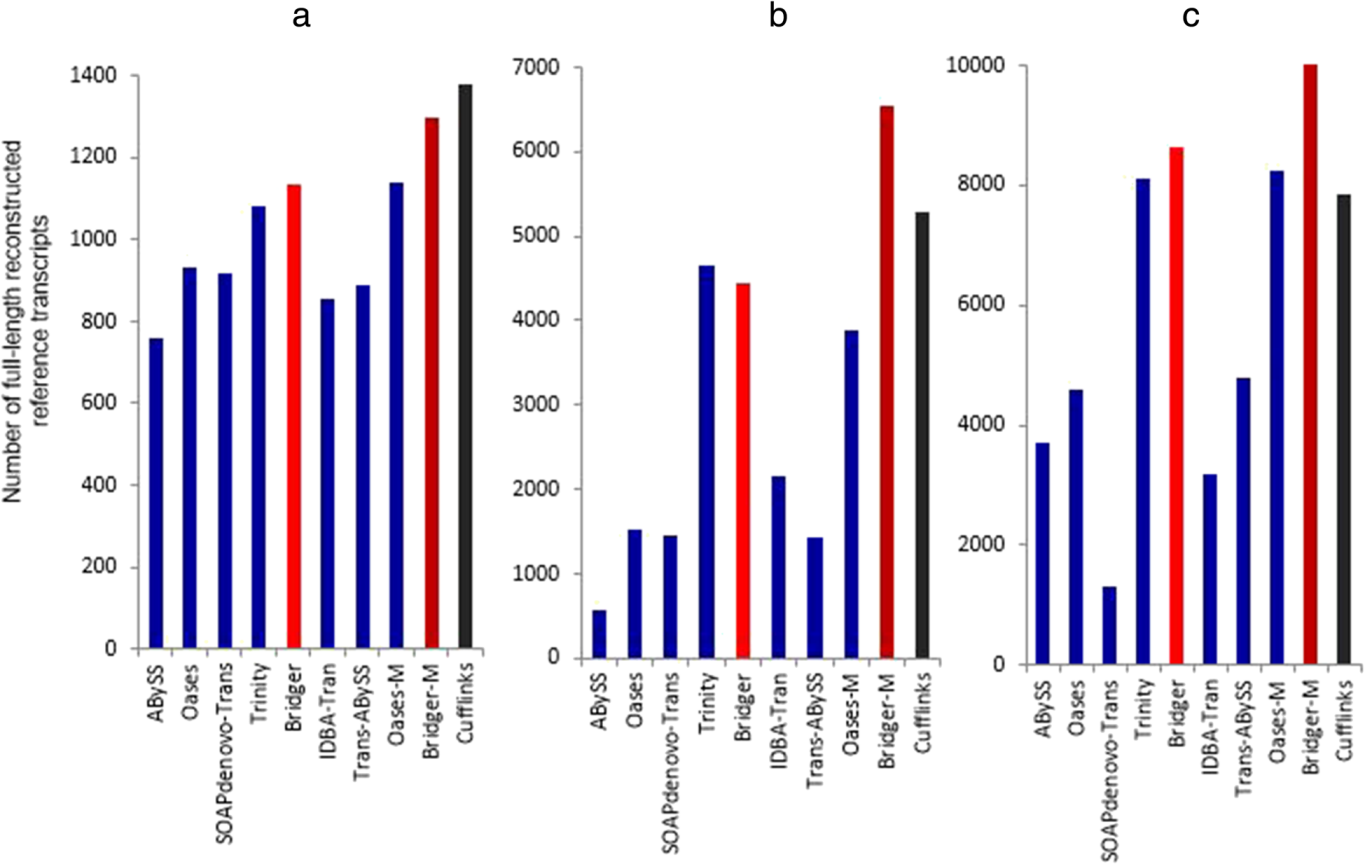
**Comparison of the number of full-length reconstructed reference transcripts for (a) dog, (b) human, and (c) mouse.**

For the sensitivity measure, Bridger outperforms all other *de novo* assemblers on all of three test data (Figure 3). Bridger-M is even better than the best reference-based assembler Cufflinks on human and mouse data (Figure 3a and c), and comparable to Cufflinks on dog data (Figure 3b). Trinity, a brute enumeration approach which should be highly sensitive, only performs a little better than Bridger on human data, but much worse than Bridger on dog and mouse data. Oases-M performs worse than Bridger, but considerably better than ABySS, Oases, SOAPdenovo-Trans, Trans-ABySS, and IDBA-Tran. Note that more significant results can be found when checking the number of ≥80% length reconstructed reference transcripts (Additional file 1: Tables S1, S2, and S3).

It is worth mentioning that Bridger reports 10,000 to 30,000 fewer candidate transcripts than most assemblers (Additional file 1: Tables S1, S2, and S3), while exhibiting high sensitivity. To show this advantage, we define accuracy measure to see which assembler detects the most reference transcripts by the least candidate transcripts.

**Figure 4:**
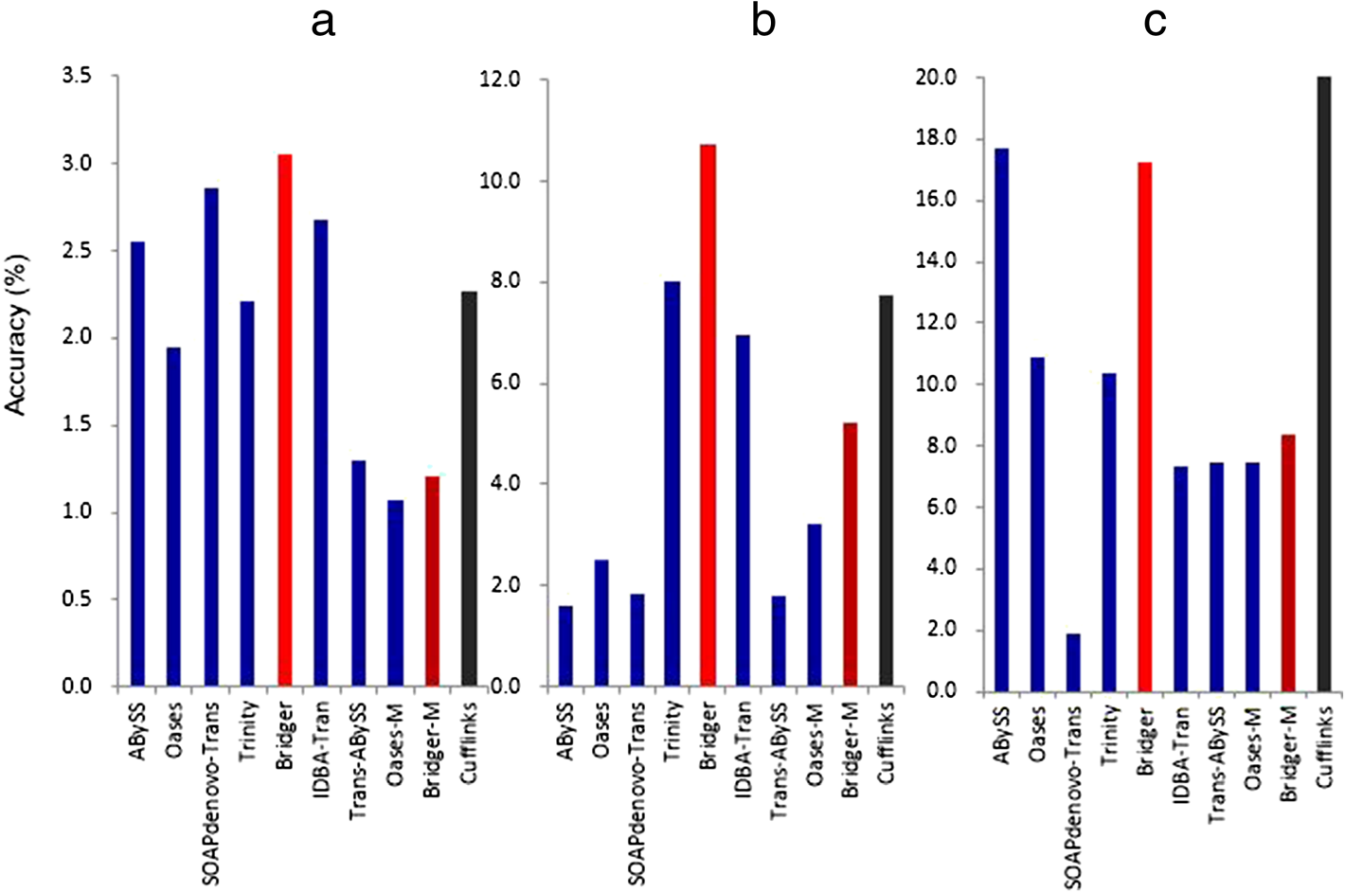
**Comparison of accuracy for (a) dog, (b) human, and (c) mouse.**

For the accuracy measure, Bridger has the highest accuracy among all *de novo* assemblers on all of three test data (Figure 4). Surprisingly, Bridger even exhibits better accuracy on dog and human data (Figure 4a), and comparable performance on mouse data (Figure 4b and c) in comparison with Cufflinks. Trinity is much worse than Bridger in the accuracy measure, indicating that its predictions contain many false positive transcripts. Oases and SOAPdenovo-Trans are comparable with or even worse than Trinity. ABySS performs well on mouse data because it reports a very small set of candidate transcripts, but it is much worse than Bridger on dog and human data. Bridger-M has much better accuracy than other multiple-*k* assemblers such as Trans-ABySS, IDBA-Tran and Oases-M, but does not exhibit better performance than Bridger, one important reason is that merging assemblies from different *k* values will introduce redundancy.

**Figure 5:**
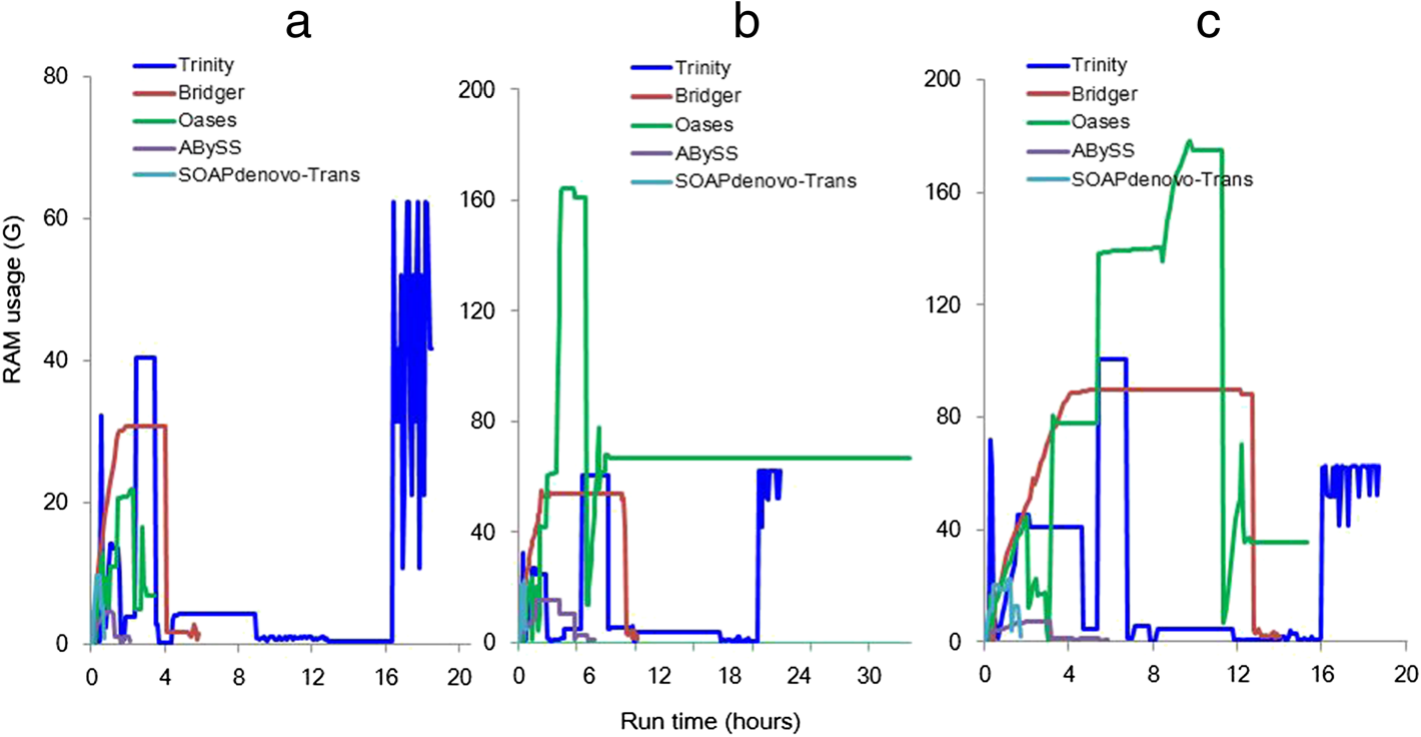
**Run time and RAM usage for each assembler in (a) dog, (b) human, and (c) mouse.** Same parameter values are used for all assemblers: k = 25 and CPU = 6.

**Figure 6:**
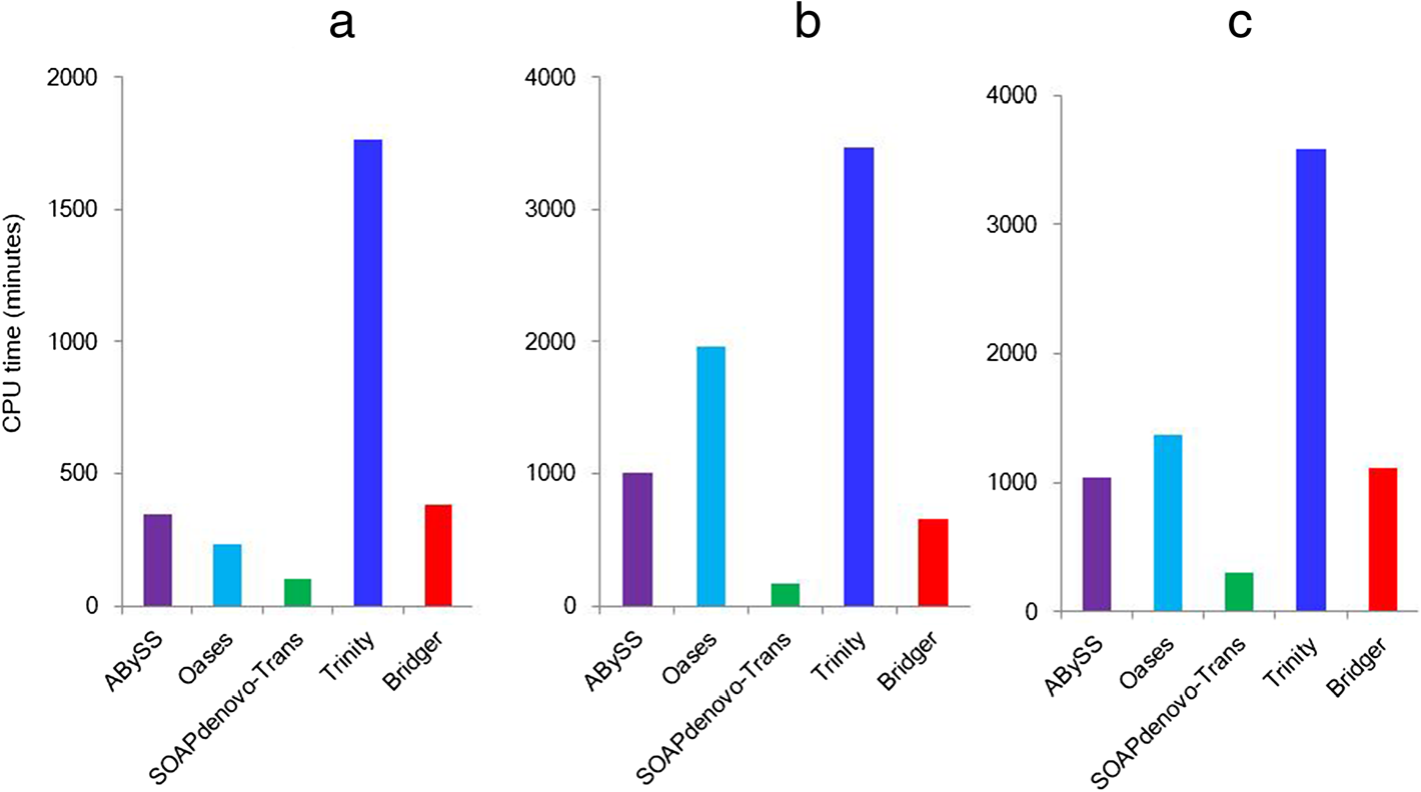
**CPU time for each assembler in (a) dog, (b) human, and (c) mouse.**

We have also examined the computing resources required, including the run time, the CPU time, and the memory usage, for the five single-*k* assemblers on the same server. The dynamic memory usage of each assembler is given in Figure 5. Oases performs well on dog data, but consumes the largest memory on both human and mouse data, and also takes the longest run time on the human data. Trinity takes the longest run time on both the dog and mouse data. Bridger requires less memory and much shorter run time than Trinity and Oases (Figure 5), especially on human and mouse data. Though ABySS and SOAPdenovo-Trans uses the smallest memory and the shortest run time, taking its worst sensitivity into account, they are not a good choice for *de novo* transcriptome assembly. For the CPU time (Figure 6), SOAPdenovo-Trans is the best; Bridger and ABySS also performs well; Trinity needs the longest CPU time on all three real data; and the CPU time needed by Oases has a distinct pattern on different datasets.

### New information for dog genome annotation

**Figure 7:**
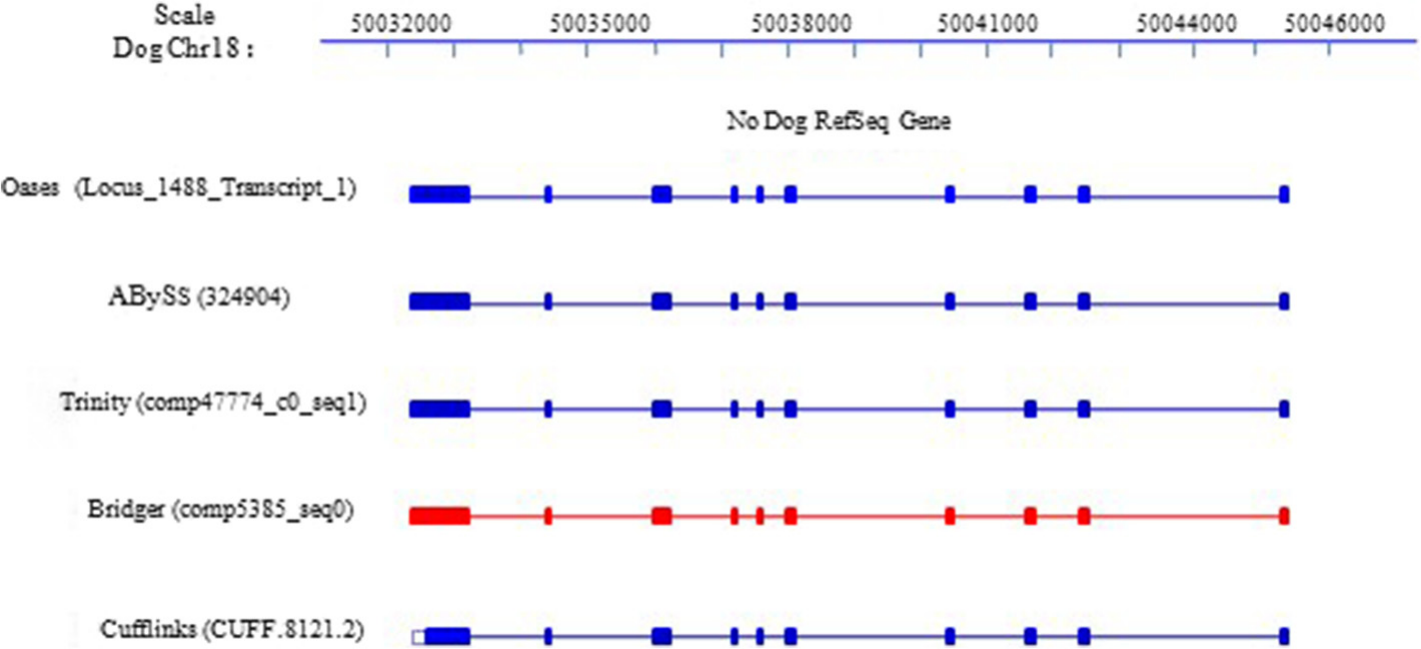
**A novel gene containing 10 exons was assembled by all assemblers.** Interestingly, all *de novo* assemblers captured longer UTR than the reference-based assembler Cufflinks.

On the dog dataset, Bridger reported 37,234 transcripts, 15,437 of which are equal or longer than 1,000 bps. By mapping all transcripts to the dog genome using BLAT and 95% identity as cutoff, we noted that 2,683 transcripts in 2,629 loci were not previously annotated. Interestingly 78.6% (2110/2683) of these novel transcripts were also predicted by at least one another assemblers under consideration; hence we consider them as possibly true. In addition, Bridger tends to predict longer un-translated regions (UTRs) than the probably most accurate assembler out there, Cufflinks (Figure 7).

## Conclusions

We present a new *de novo* method Bridger for transcriptome reconstruction from short RNA-seq reads. Trinity has been the best *de novo* assembler using one single *k* value since it was released in 2011. Though several multiple-*k* assemblers have a little higher sensitivity than Trinity, they all suffer from the problem of introducing much more false positive transcripts. Bridger is a single-*k de novo* assembler that outperforms Trinity in both sensitivity and accuracy. Compared to Trinity, Bridger has the following advantages: (1) Trinity uses a fixed *k*-mer length 25, which is not necessarily optimal for all data, while Bridger allows using different user-specified *k* values for different data; (2) Bridger uses a rigorous mathematical model to search for a minimum set of paths from the splicing graph as in Cufflinks compared to the nearly exhaustive search method used in Trinity, which gives Bridger a lower false positive rate at the same level of sensitivity; (iii) Bridger successfully incorporate the sequence-depth information, which is implicitly considered in the node weights of the compatibility graphs, to constrain the deconvolution of splicing graphs into individual transcripts, hence making its assembly results more accurate; (4) Bridger utilizes paired read information to help reconstruct more complete splicing graphs, making contigs even not covered by overlapping *k*-mers recovered during assembly - the similar technique can also be found in a recent assembler SOAPdenovo-Trans [22]; (5) Bridger is a polynomial-time algorithm, while Trinity is an exponential-time method. In practice, Bridger uses less memory and much less run time compared with Trinity.

Minimum path cover model has been used in reference-based assembler Cufflinks [12], and also been applied to a recent reference-assisted method called BRANCH [32], which need use genomic information that can be partial or complete genome sequences from the same or related organism. Bridger successfully uses this strategy for *de novo* transcriptome assembly by introducing an auxiliary graph, without the help of any genomic information. When tested on three real data, Bridger shows best sensitivity and accuracy among all *de novo* assemblers, and even comparable to the reference-based assembler Cufflinks. In addition, it requires much less computational resources than other *de novo* assemblers, except ABySS, which is actually a genome assembler and hence has the worst performance. With these demonstrated advantages, we anticipate that Bridger will play an important role for new discovery in transcriptome study using RNA-seq data, especially for cancer transcriptomic data analyses.

## Additional file

Additional file 1:
**This file includes details of Bridger method, discussion about optimizing** ***k*** **-mer length, plus supplementary figures and tables.**

## Abbreviations

DDBJ: DNA Data Bank of Japan
EST: Expressed sequence tag
NCBI: National Center for Biotechnology Information
RNA-seq: RNA sequencing
SRA: Sequence read archive
UTR: Untranslated region
