# Supplementary material for: Bridger: a new framework for de novo transcriptome assembly using RNA-seq data
Source: Genome Biol. 2015 Feb 11;16(1):30. doi: 10.1186/s13059-015-0596-2 (PMC4342890; doi:10.1186/s13059-015-0596-2)
Supplement: Additional file 1: — This file includes details of Bridger method, discussion about optimizing k -mer length, plus supplementary figures and tables. [file 13059_2015_596_MOESM1_ESM.docx]

SUPPLEMENTARY MATERIAL FOR THE PAPER

**Bridger: A new framework for *de novo* transcriptome assembly using RNA-seq data**

Zheng Chang^1,†^, Guojun Li^1,†,*^, Juntao Liu^1^, Yu Zhang^6^, Cody Ashby^4,5^, Deli Liu^2,3^, Carole L. Cramer^4^, Xiuzhen Huang^4,5,*^

1. **SUPPLEMENTARY METHODS**

**Bridger assembles splicing graphs greedily and efficiently**

We first build a hash table from all the reads. For each *k*-mer (default *k*=25) occurring in the reads, the hash table records the abundance of that *k*-mer and the IDs of reads containing that *k*-mer. To reducing memory usage, each*k*-mer is stored as a 64-bit unsigned integer with 2-bit nucleotide encoding, thus the parameter *k* is not allowed to be larger than 32. Then, we remove error-containing *k*-mers and select seed *k*-mers by the same strategy in Trinity[[1](#_ENREF_1)]. A *k*-mer chosen as a seed must meet the following criteria: (a) Shannon’s entroy [[2](#_ENREF_2)] of the *k*-mer H$\geq$ 1.5, (b) the *k*-mer occurs at least twice in the complete set of input reads, and (c) the *k*-mer is not palindromic [[1](#_ENREF_1)]. The seed *k*-mer is extended to a complete splicing graph greedily in the following steps:

(1) We extendthe seed *k*-mer in two directions by repeatedly selecting the most frequent *k*-mer in the hash table, with *k*-1 overlaps with the current contig terminus, in order to provide a single-base extension. Those *k*-mers used for extension are marked to indicate their lower priority of being reused for extension in the future.

(2) When the contig cannot be extended, we use paired-read information to get further extension. Based on our hash table, the reads mapping to the terminus of this contig are easily collected. If some of their paired-reads are not used in the current splicing graph, which implies the contig is not complete. A new contig can be generated from those unused end of paired-reads, and then connected to existing contig by using pair information (Fig. S1).Thus, some transcripts which cannot be covered by overlapping k-mers can be reconstructed. The ultimate contig is used as the trunk of a splicing graph.

(3) We check each k-mer in the trunk to see if there exists a k-mer having an alternative extension that has not been used (such a k-mers is called a bifurcation k-mer). Once a bifurcation k-mer is found, we extend it to a contig as long as possible.

(4)If this contig can be extend by some used *k*-mer in current graph, we can identify a new bifurcation *k*-mer and modify current splicing graph by merging *k*-1 overlapping nucleotides and adding one directed edge between them (Figure 3). Otherwise, the following criteria are used to check if this potential branch is allowed to add to current splicing graph: (i) the branch is long enough (>=80 bp) to be an exon (Fig. S2a); (ii) the branch is not as same as the corresponding part of the trunk (Fig. S2b); (iii) there are at least two read pairs supporting this branch (Fig. S2c).

Two paralogous genes can be separated by using paired read information. One example is shown in Fig. S2d. The new branch will be not added into current graph if we find a paired read with one end (the black one) mapping to the branch and the other end (the green end) mapping to outside of the current graph (note that the red dash does not exist in current splicing graph). When we construct the splicing graph of the red gene, the “hole” resulting from the first gene (blue gene) can be filled by used k-mers (The used k-mers are only allowed to be reused to fill such holes).

(5)We grow the splicing graph by repeatedly finding bifurcation k-mers, until no bifurcation k-mer exists.

(6) We mark all used *k*-mers and trim edges induced from sequencing errors by the similar criteria used in Trinity: (a) for each edge, there is a minimal number of reads(default 2) perfectly match at least (*k*-1)/2 bases on each side of the junction. (b)The average k-mer coverage of each edge must exceed 0.04 times the average k-mer coverage of two franking nodes(twice the sequencing error rate in a read, the upper bound is about 2%).(c) If there is one node with several outgoing edges, each one of them should have a read support more than 5% of the total outgoing reads. (d) Any outgoing edge has a support more than 2% of the total incoming reads. Edges in splicing graph that does not meet any one of these criteria are removed.

Splicing graphs with less than minimum number of k-mers are discarded (an empirical value used by Trinity is 300-(*k*-1) =276). For non-strand specific RNA-seq data, both *k*-mer and the reverse-complemented *k*-mer are considered in building the hash table, extending the splicing graph. A splicing graph is a compacted directed acyclic graph, and ideally each node, which is a fragment of sequence, corresponds to one exon and each edge represents one junction.

**Bridger constructs weighted directed acyclic compatibility graphs**

In a splicing graph, nodes correspond to the exons and edges represent splice junctions. One transcript is one path of the splicing graph, but not every path in the splicing graph is necessarily one real spliced transcript (Fig. S3a), especially for complicated splicing graphs. Our goal here is to obtain a set of transcripts meet the following criteria: (a) each junction of the splicing graph can be explained by at least one transcript; (b) every transcript is tiled by sequence reads; (c) the cardinality of obtained set of transcripts is minimized subject to (a) and (b). To this end, the minimum path cover model used by Cufflinks [[3](#_ENREF_3)] is promising to be used here. However, one challenge is that we want to obtain a set of paths that could cover all edges (junctions), instead of only covering all nodes in splicing graph (Fig. S4).Thus, we first construct an auxiliary graph, and then apply minimum path cover model to this new graph. Two consecutive edges in splicing graph are compatible if they could originate from a same spliced isoform. Based on this, a directed graph *C*, called compatibility graph (Fig. S3b) is constructed as follows: each edge (junction) of splicing graph is assigned as one node of *C*, a directed edge (*x*, *y*) was placed between nodes *x* and *y* if they were compatible. The compatibility graph defined above could play the same role as the overlap graph in Cufflinks, so we would recover all the full-length transcripts by employing the techniques in Cufflinks over the overlap graph.

**Bridger resolves full-length transcripts**

Finding minimum path cover in a directed acylic graph (DAG) is well defined and has a polynomial-time algorithm. A partial order (Definition 1) is constructed from the transitive closure *G* (Fig. S3c) of compatibility graph *C* by declaring that *x*$\leq$*y* whenever there exists edge (*x, y*) in *G*. By Dilworth’s theorem (Theorem 1) [[4](#_ENREF_4)], finding a minimum path cover is equivalent to finding a maximum antichain (an antichain here is a set of mutually incompatible nodes in *G*). In next section (Algorithm MPC), we will prove that finding a maximum antichain can be reduced to finding a maximum matching of a certain bipartite graph, called the reachability graph (Fig. S3d), which is constructed from *G*. For each node *x* in *G*, we have *L_x_* and *R_x_* in the left and right partitions of the reachability graph respectively, and there is an edge between *L_x_* and *R_y_* if there is an directed edge (*x, y*) in *G*.

However, the minimum path cover computed in this way is not guaranteed to be unique, so we add weights to this model in a way similar to Cufflinks. First, we assign each edge (*e’, e’’*) of the splicing graph two weights, out-weight *W_o_* , whichis computed by counting the number of reads (or paired reads for paired-end sequencing) spanning the junction, dividing by the total number of reads spanning all junctions that share the same 5’-end exon as the junction (including itself)_,_ and in-weight *W_i_*_,_ which is computed by counting the number of reads (or paired reads for paired-end sequencing) spanning the junction, dividing by the total number of reads spanning all junctions that share the same 3’-end exon as the junction (including itself). The number of reads spanning one junction could be approximately calculated by the average *k*-mer coverage of a fragment of sequence spanning the junction with (*k*-1)/2 bases match on each side of the junction. Then, out-weight and in-weight of each node of the compatibility graph are defined as out-weight and in-weight of the corresponding edge in the splicing graph. The weigh between nodes *L_x_* and *L_y_* in the reachability graph, *W_x,y_*, which reflects the belief that they originated from different transcripts, is defined as: *W_x,y_= -log(1 - |W_x,i_ - W_y,o_ |)*, where *W_x,i_* and *W_y,o_* are in-weight of node *x* and out-weight of node *y* in the compatibility graph *C*. If two junctions coming from the same transcripts, they should have similar expression level (coverage), so a small weight should be assigned between them. We use a modified version of LEMON(<http://lemon.cs.elte.hu/trac/lemon>) and Boost(<http://www.boost.org/>)graph libraries to find a min-cost maximum cardinality matching on the bipartite reachability graph. Although the best known algorithm for weighted maximum matching is *O(|V |^2^ log |V |+ |V ||E |)*, our algorithm is very fast in practice due to the small size of the graph.

Given a min-cost maximum cardinality matching M, any node without an incident edge in M is a member of an ‘antichain’. Each member of this antichain could be extended to a path by using M, which will be further extended if it does not correspond to a full-length transcript (see Fig. S3d). The set of paths obtained is a min-cost minimum path cover of the compatibility graph, which can be easily converted into a set of paths of the splicing graph. Of course, paired reads, if available, could be used to filter some false positive transcripts. For each assembled transcript, we require at least two read pairs supporting the combination of two consecutive exons (Fig. S3e).

**Definition1**A partially ordered set is a set *S* with a binary relation $\leq$satisfying:

(1) *x*$\leq$*x* for all *x*$\in$*S*,

(2) If *x*$\leq$*y*and *y*$\leq$*z* then *x*$\leq$*z*,

(3) If *x* $\leq$*y*and *y*$\leq$x then *x* = *y*.

A chain is a set of elements S’S such that for every *x, y*$\in$S’ either x $\leq$ y or y $\leq$x. An antichain is a set of elements that are pairwise incompatible.

**Theorem1** (Dilworth’s theorem,1950[[4](#_ENREF_4)]) Let *P* be a finite partially ordered set. The maximum number of elements in any antichain of *P* equals the minimum number of chains in any partition of P into chains.

**Algorithm for Minimum Path Cover (MPC)**

Given a DAG *G =* (*V, A*) with vertex set *V* = {*1,…,n*}. Construct a bipartite graph *G’ =* (*V*$\cup$*V’, E*), where*V’* = {*1’,…,n’*} and {*v, w’*}$\in$*E* if and only if (*v, w*)$\in$*A*.The minimum path cover *P* of *G* could be reconstructed from a maximum matching *M** of *G’* as follows:

Algorithm MPC

1 *P*=$\emptyset$

2 Repeat until *P* covers every vertex of *G*

3 Choose any *v*$\in$*V*, *s.t*. *v*∉*P* and *v’**M**

4 p = GrowAPath(*v*)

5 *P* = *P* $\cup${*p*}

6 Return P

Procedure GrowAPath(*v*)

1 *p*= {*v}*

2 While *v* is matched to some vertex *w’*

3 *p*= p$\cup${*w*}

4 *v* =*w*

5 Return p

The algorithm MPC is well defined—there always exists a vertex *v* to choose at step 3 until *P* is a path cover, which depends on the acyclicity of *G*. Here we prove that the path cover output by MPC must be minimum. Let |*M**| = *m**. We claim first that P has *n-m** paths. In fact, the number of paths in *P* is the number of “starting nodes” *v* on which we called GrowAPath. We called GrowAPath (*v*) if and only if *v’* was unmatched, implying that the number of such starting nodes *v* is the number of unmatched vertices in *V’*, which is *n-m**.Now we prove there does not exit a smaller path cover. Assume to the contrary that there exists such a path cover with *k (<n-m*)* paths. Then *G’* has a matching with *n-k* edges. Since *k* < (*n-m**), there exists a matching with more than *m** edges (*n-k* >*n*-(*n-m**) = *m**), contradicting that *M** is maximum matching. What is left to be shown is that if G has a path cover with *k* paths, then *G’* has a matching with *n-k* edges. In fact, we could construct a matching *M* as follows: {*v, w’*} $\in$*M* if and only if *v*🡪*w* lies along one path of the path cover. This is a matching of *G’* since any vertex *v* is matched to at most one *w’* or any vertex *w’* is matched to at most one *v*. Every vertex *v*$\in$*V* is either the initial point of a path or an internal point pointed by a unique edge of a path, so *n* = *k*+|*M*| and so |*M*| = *n* - *k*, proved.

1. **SUPPLEMENTARY NOTE**

**Optimizing *k*-mer length of Bridger**

One crucial parameter in Bridger is the *k*-mer length. Generally speaking, larger *k* values perform best on high expression data or longer reads and smaller *k* values perform best on low expression data or shorter reads. Comparing the assemblies in terms of different *k* (Fig.S5), we observed that *k*=19 or less is bad for all data; *k*=25 is the best for dog and human data, but not for mouse data; *k*=31 is the best for mouse data (Table S4, S5, S6). In current version of Bridger, *k*=25 is chosen to be the default *k* value, however, larger *k* is recommended for reads with length longer than 75bp (like mouse data in our study).

**Optimizing parameters of other de novo assemblers**

The default parameters are always used for all the assemblers except that there exist better settings for them, which are specified here. For Oases and ABySS, we ran them multiple times on the mouse data in order to obtain an optimal parameter *k* for both of them (Fig. S6).The results indicated that 25 is not the optimal value for the parameter *k*. Instead, 31 and 33 are the best choice for Oases and ABySS respectively. Non-default parameters such as “-cov_cutoff 2 ‑edgeFractionCutoff 0.05” are used for Oases because it results in better performance than the default parameters (see Table S7).There is no knowledge about how to select the *k* range for multiple-k assemblers. Generally speaking, larger *k* values tend to perform better on transcripts with high gene-expression levels or longer reads, while smaller *k* values perform better on transcripts with low gene-expression levels or shorter reads. Based on this, and also according to one recent comparison study of different *de novo* transcriptome assemblers [5], we choose the *k* range to be 21, 25, 29, 33, 37on dog and human for multiple assemblers Trans-ABySS, Oases-M and IDBA-Tran. Because the read length of mouse data is much larger than that of dog and human data, so the k range is set to be 25, 29, 33, 37 and 43 to get a better performance (see Table S8). For Bridger-M, which does not allow *k* value greater than 32, we choose the *k* range to be 21, 23, 25, 27, 29 on dog and human data, and 23, 25, 27, 29, 31 on mouse data. Other parameters which are not mentioned here are kept their default settings.

1. **Supplementary Figures**


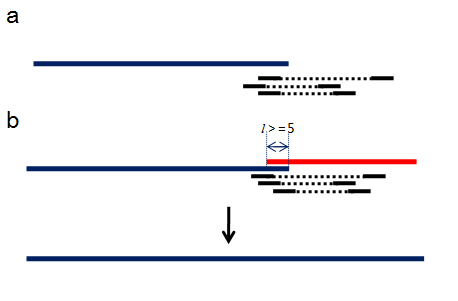


**Figure S1**. Paired read information is used for constructing a complete trunk of the splicing graph. When the contig cannot be extended by overlapping *k*-mers, Bridger (a) collects all paired-end reads with one end mapping the terminus of the contig and the other end mapping outside and (b) generates a new contig starting from the end mapping outside of the current contig. Then these two contigs can be connected into a longer one.

**
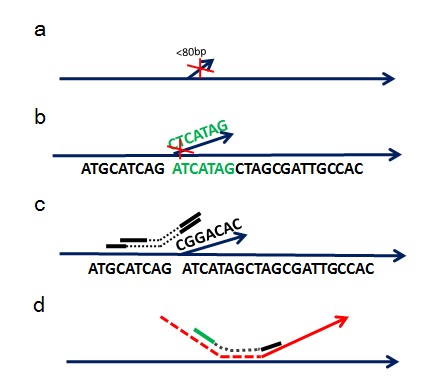
**

**Figure S2**. Criteria used to decide if one potential branch is allowed to be added into the current splicing graph. (a) A branch must be long enough. If not, ignore it. (b) A branch must be different from the corresponding part of the trunk. If not, ignore it. (c) A branch that meets (a) and (b) is allowed to be added into the graph if there exist at least two paired-end reads supporting it. (d) Two paralogous genes, colored with red and blue respectively, can be separated by paired read information.


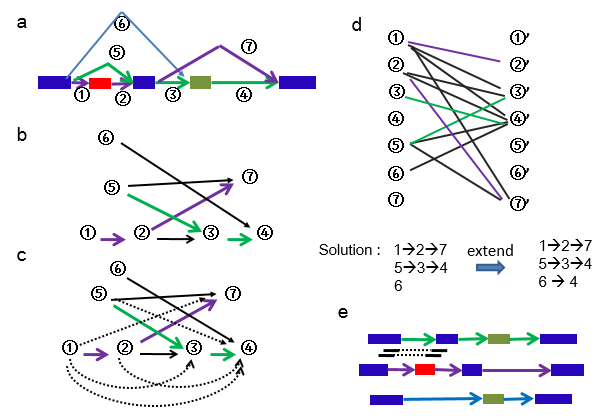


**Figure S3**. One example showing how minimum path cover model is used to resolve transcripts. (a) Splicing graph. There are five possible paths, but only three paths (1->2->7, 5->3->4 and 6->4) are real transcripts. (b) Compatibility graph, with nodes correspond to edges of the splicing graphs, and edges are added to each pair of the compatible nodes. (c) Transitive closure *G* of the compatibility graph. (d) Reachability graph constructed from *G*. A path cover can be obtained from the maximum matching of reachability graph. Note that transcripts in this path cover will be further extended if they are not full-length so that different transcripts sharing the common junction could be constructed. (e) Paired-end reads are used to filter some false positive transcripts. Those transcripts that are not supported by tiled paired-reads with coverage at least 2 are considered as false positive and would be removed.

**
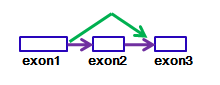
**

**Figure S4.** One example illustrating transcriptome reconstruction is to find a set of paths that could cover all edges (junctions), instead of all nodes in the splicing graph. In this example, one path exon1->exon2->exon3 can cover all nodes in this graph, but obviously, there exists another transcript exon1->exon3.


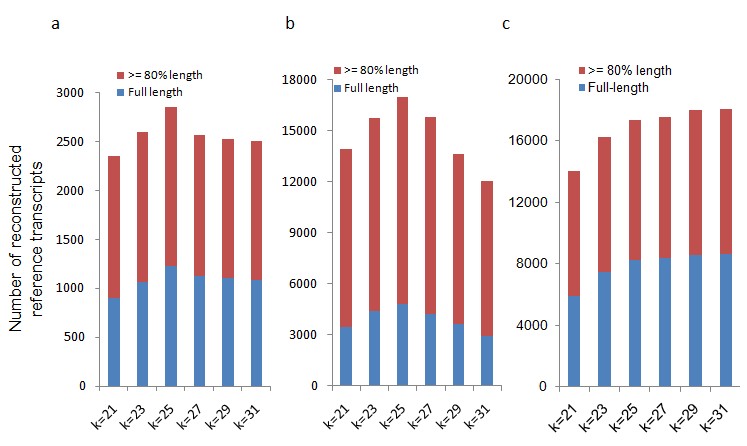


**Figure S5**. Analysis of assemblies from different *k* values for Bridger on (a) dog, (b) human and (c) mouse (c). Both full length reconstructed reference transcripts and >=80% length reconstructed reference transcripts are shown.


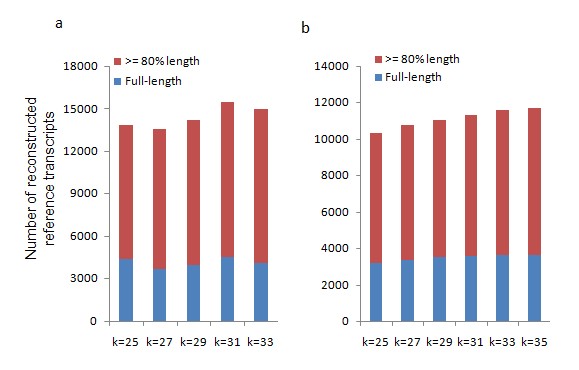


**Figure S6**. Analysis of parameter *k* for Oases and ABySS on mouse data. Both (a) Oases and (b) ABySS show *k*=25 is not optimal, consistent with the results of Bridger (*k*=31 is optimal for Oases and Bridger, *k*=33 is optimal for ABySS).


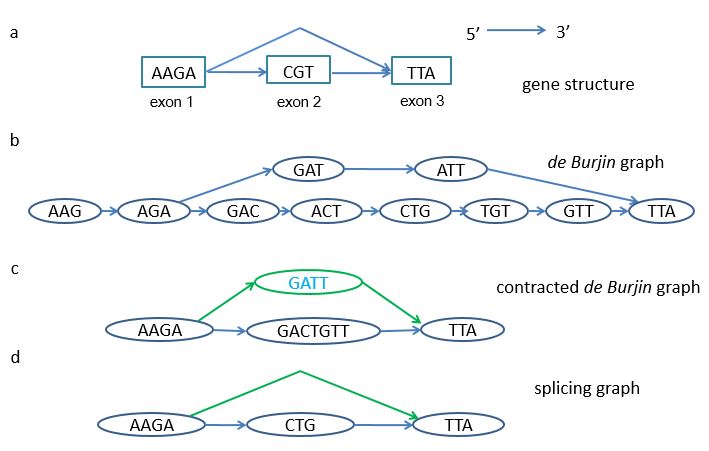


**Figure S7**. One example shows that the splicing graph is different from contracted *de Bruijn* graph. (a) Gene structure with two isoforms. (b) *De Bruijn* graph. (c) Contracted *de Bruijn* graph. (d) Splicing graph.

1. **SUPPLEMENTARY tables**

Table S1. Comparison of different RNA-seq assembly methods on dog.

| Method | #Candidate  transcripts | Full-length reconstructed reference transcripts | >= 80% length reconstructed reference transcripts | |
| --- | --- | --- | --- | --- |
| ABySS | 29842 | 760 | | 2119 |
| Oases | 47896 | 934 | | 2406 |
| SOAPdenovo-Trans | 32057 | 916 | | 2015 |
| Trinity | 49031 | 1082 | | 2553 |
| Bridger | 37234 | 1135 | | 2642 |
| IDBA-Tran | 32057 | 857 | | 2379 |
| Trans-ABySS | 68283 | 887 | | 2496 |
| Oases-M | 106231 | 1140 | | 2956 |
| Bridger-M | 107522 | 1298 | | 3255 |
| Cufflinks | 60814 | 1380 | | 10984 |

Table S2. Comparison of different RNA-seq assembly methods on human.

| Method | #Candidate  transcripts | Full-length reconstructed reference transcripts | >= 80% length reconstructed reference transcripts | |
| --- | --- | --- | --- | --- |
| ABySS | 36132 | 573 | 4291 |  |
| Oases | 60363 | 2521 | 9974 |  |
| SOAPdenovo-Trans | 80455 | 1462 | 8517 |  |
| Trinity | 58315 | 4662 | 16160 |  |
| Bridger | 41470 | 4441 | 16094 |  |
| IDBA-Tran | 31095 | 2155 | 13373 |  |
| Trans-ABySS | 79070 | 1432 | 10634 |  |
| Oases-M | 121372 | 3677 | 17942 |  |
| Bridger-M | 125510 | 6553 | 21436 |  |
| Cufflinks | 68067 | 5272 | 18387 |  |

Table S3. Comparison of different RNA-seq assembly methods on mouse.

| Method | #Candidate  transcripts | Full-length reconstructed reference transcripts | >= 80% length reconstructed reference transcripts | |
| --- | --- | --- | --- | --- |
| ABySS | 20334 | 3699 | 11644 |  |
| Oases | 42104 | 4597 | 15538 |  |
| SOAPdenovo-Trans | 110830 | 1313 | 9626 |  |
| Trinity | 78333 | 8126 | 17238 |  |
| Bridger | 50018 | 8624 | 18038 |  |
| IDBA-Tran | 43717 | 3198 | 11266 |  |
| Trans-ABySS | 64317 | 4780 | 13704 |  |
| Oases-M | 110574 | 8235 | 18758 |  |
| Bridger-M | 129264 | 10802 | 20706 |  |
| Cufflinks | 25108 | 7858 | 16662 |  |

Table S4. Analysis of Bridger assemblies for different k values on dog.

| *k* value | #Candidates transcripts | Full-length reconstructed reference transcripts | >=80% length reconstructed reference transcripts |
| --- | --- | --- | --- |
| k=21 | 45959 | 905 | 2358 |
| k=23 | 40519 | 1103 | 2611 |
| k=25 | 37234 | 1135 | 2942 |
| k=27 | 34875 | 1135 | 2599 |
| k=29 | 32779 | 1110 | 2560 |
| k=31 | 31003 | 1100 | 2540 |

Table S5. Analysis of Bridger assemblies for different *k* values on human.

| *k* value | #Candidates transcripts | Full-length reconstructed reference transcripts | >=80% length reconstructed reference transcripts |
| --- | --- | --- | --- |
| k=21 | 49622 | 3455 | 13934 |
| k=23 | 45172 | 4398 | 15799 |
| k=25 | 41470 | 4441 | 16094 |
| k=27 | 38119 | 4252 | 15816 |
| k=29 | 34981 | 3635 | 13657 |
| k=31 | 31912 | 2974 | 12122 |

Table S6. Analysis of Bridger assemblies for different k values on mouse.

| *k* value | #Candidates transcripts | Full-length reconstructed reference transcripts | >=80% length reconstructed reference transcripts |
| --- | --- | --- | --- |
| k=21 | 62573 | 5902 | 14058 |
| k=23 | 58891 | 7452 | 16232 |
| k=25 | 56448 | 8225 | 17331 |
| k=27 | 54857 | 8365 | 17505 |
| k=29 | 52384 | 8589 | 17956 |
| k=31 | 50018 | 8624 | 18038 |

Table S7. Analysis of Oases assemblies with default or non-default parameters.

| Data | Parameters | Full-length reconstructed reference transcripts | >=80% length reconstructed reference transcripts |
| --- | --- | --- | --- |
| dog | default | 902 | 2362 |
|  | non-default | 934 | 2406 |
| human | default | 1369 | 9432 |
|  | non-default | 1521 | 9974 |
| mouse | default | 4078 | 13815 |
|  | non-default | 4597 | 15538 |

Table S8. Analysis of multiple-k assemblers with different k range on mouse data.

| Assembler | K range | Full-length reconstructed reference transcripts | >=80% length reconstructed reference transcripts |
| --- | --- | --- | --- |
| Trans-ABySS | 21,25,29,33,37 | 4565 | 12261 |
|  | 25,29,33,37,43 | 4780 | 13704 |
| Oases-M | 21,25,29,33,37 | 8016 | 16852 |
|  | 25,29,33,37,43 | 8235 | 18758 |
| IDBA-Tran | 21,25,29,33,37 | 2900 | 10762 |
|  | 25,29,33,37,43 | 3198 | 11266 |

**References**

1. Grabherr MG, Haas BJ, Yassour M, Levin JZ, Thompson DA, Amit I, Adiconis X, Fan L, Raychowdhury R, Zeng Q: Full-length transcriptome assembly from RNA-Seq data without a reference genome. Nature biotechnology 2011, 29:644-652.

2. Shannon CE: Prediction and entropy of printed English. Bell system technical journal 1951, 30:50-64.

3. Trapnell C, Williams BA, Pertea G, Mortazavi A, Kwan G, van Baren MJ, Salzberg SL, Wold BJ, Pachter L: Transcript assembly and quantification by RNA-Seq reveals unannotated transcripts and isoform switching during cell differentiation. Nature biotechnology 2010, 28:511-515.

4. Dilworth RP: A decomposition theorem for partially ordered sets. The Annals of Mathematics 1950, 51:161-166.

5. Zhao Q-Y, Wang Y, Kong Y-M, Luo D, Li X, Hao P: Optimizing de novo transcriptome assembly from short-read RNA-Seq data: a comparative study. BMC bioinformatics 2011, 12:S2.
